# Competency-Based Assessment in Experiential Learning in Undergraduate Pharmacy Programmes: Qualitative Exploration of Facilitators’ Views and Needs (ACTp Study)

**DOI:** 10.3390/pharmacy10040090

**Published:** 2022-07-26

**Authors:** Sabrina Anne Jacob, Ailsa Power, Jane Portlock, Tesnime Jebara, Scott Cunningham, Anne C. Boyter

**Affiliations:** 1 Strathclyde Institute of Pharmacy and Biomedical Sciences, Glasgow G4 0RE, UK; sabrina.jacob@strath.ac.uk; 2 NHS Education for Scotland, Glasgow G3 8BW, UK; ailsa.power@nhs.scot; 3 School of Life Sciences, University of Sussex, Brighton BN1 9QG, UK; j.c.portlock@sussex.ac.uk; 4 School of Pharmacy & Life Sciences, Robert Gordon University, Aberdeen AB10 7GJ, UK; t.jebara1@rgu.ac.uk (T.J.); s.cunningham@rgu.ac.uk (S.C.)

**Keywords:** experiential learning, pharmacy, facilitators, student pharmacist, competency-based assessment

## Abstract

Newly registered pharmacists will need to possess higher-level competencies and, in Great Britain, there is an expectation that assessments are undertaken during experiential learning (EL). The aim of this study was to explore the perceptions and educational needs of practice-based EL facilitators of student pharmacists, undertaking competency-based assessments during EL. Semi-structured one-on-one interviews were conducted with EL facilitators working in the community, hospital, and primary-care pharmacies. Data were thematically analysed. Fifteen facilitators were interviewed, and there were five from each site. There was general support for this role, but also anxiety due to the lack of knowledge about assessments and the repercussions on students. Benefits were that students would receive real-time feedback from workplace-based practitioners and facilitators would benefit from self-development. Challenges included additional workload and lack of consistency in marking. The majority agreed that clinical, professional, and communication skills could be assessed; however, a consensus was not reached regarding the tools, methods, and grading of assessments. The need for training and support were highlighted. A co-design method was proposed to ensure that the assessment methods and processes are accepted by all stakeholders. Training and resources should be tailored to the needs of facilitators.

## 1. Introduction

The primary goal of health professional education is to produce healthcare professionals who are able to provide effective patient-centred care and meet the demands of the dynamic practice setting [1,2]. To align with this, Schools of Pharmacy are increasingly moving away from a traditional input-based approach to competency-based education, as simply possessing good knowledge does not necessarily ensure student pharmacists will become competent practitioners [3]. In competency-based education, the focus is instead on the ability of student pharmacists to use and apply their skills and knowledge to perform their roles as pharmacists, and to meet the demands of the population being served and the healthcare system [4,5]. 

In tandem with that, it is crucial for appropriate workplace-based assessment methods to be adopted—ones that guide learning and facilitate the development of the necessary skills and competencies expected of students in practice and as stipulated by the professional standards of the programme [6,7,8]. Indeed, assessments should not be seen as an end-point to test students’ knowledge; rather, it should be viewed as a tool to be used to identify gaps in learning so that the necessary actions can be adopted to help students achieve the outcomes desired [9,10]. Thus, there is a shift from ‘assessment of learning’ to ‘assessment for learning’ [8].

Competency-based education is an approach to teaching and assessments that aims to develop healthcare professionals who are competent enough to meet the demands of the healthcare environment [10]. Competency-based assessments (CBAs) were then introduced as a way to evaluate the competencies developed by students [1]. CBAs use data captured from the assessment process to ensure that students are provided with the appropriate feedback at the different stages and supports the practice of self-reflection, all in order to ensure that they are indeed competent to practice and provide appropriate patient care [10]. This aligns closely with Miller’s Pyramid—‘knows’, ‘knows how’, ‘shows how’, and ‘does’—as well as Kolb’s Experiential Learning cycle, which are both key tools used in experiential learning (EL) [11,12]. In the United Kingdom (UK), competencies required of pharmacists are outlined in the Initial Education and Training (IET) Standards for Pharmacists, published by the General Pharmaceutical Council (GPhC), and are assessed against Miller’s Pyramid [13].

Independent prescribing by pharmacists was implemented in the UK in 2006 to align with the government’s efforts to enhance patient care [14,15]. As independent prescribers, pharmacist are allowed to examine the patient and prescribe medication, and will therefore be held accountable for patient care [15]. The role will also require prescribers to possess and display higher-level competencies, as outlined in the Royal Pharmaceutical Society’s (RPS) competency framework for prescribers, such as undertaking patient assessment, using evidence-based treatment in clinical decision-making, and adopting a shared-decision approach [16]. Under the new Standards published in 2021, all trainee pharmacists who successfully complete their Foundation Training Year (previously ‘pre-registration year’) will be allowed to register as pharmacist prescribers from July 2026 [13]. Previously, pharmacists had to be a registered pharmacist with the GPhC or the Pharmaceutical Society of Northern Ireland (PSNI) and have at least two years’ appropriate patient-orientated experience in a UK hospital, community, or primary care setting following their pre-registration year before being able to register as a prescriber. From May 2022, the GPhC Council has agreed to make further changes to the requirements for entry to accredited independent prescribing courses. These changes will allow pharmacists to begin an independent prescriber course when they have the relevant experience and awareness, rather than simply completing a specified period.

To ensure student pharmacists have the necessary competencies required to undertake this expanded role, the Standards have an expectation that on-site assessments should be undertaken as part of quality assuring EL [13]. Indeed, as noted by Harris et al., ‘*to examine Miller’s “does” domain, assessment has to move to the workplace and incorporate authentic interactions in clinical environments*’ [10]. A nationwide survey involving all UK universities offering MPharm programmes, however, found that currently minimal amounts of assessment are undertaken during placements (39%), with the majority (59%) undertaken by university staff and on return to the university (61%) [17].

Schools of Pharmacy in Scotland are planning a redesign of EL where EL facilitators—registered, practising pharmacists who supervise student pharmacists [18]—may undertake CBAs of students during EL. This will also ensure a seamless transition into the Foundation Training Year, wherein trainee pharmacists are also assessed on their competencies by designated supervisors. [4,19]. Assessments during EL, however, comes with its own challenges, as it will require multiple observers, and each student’s learning and experience will differ depending on facilitators, placement sites, and individual learning styles, among other factors [6,10,20,21]. Thus, it is important that assessments are designed appropriately so that they are multifaceted, involve the continuous evaluation of students, encourage engagement with the practice-setting, and develop student pharmacists who can demonstrate competence in the workplace [6,7]. In addition, this is a novel approach in Pharmacy education in Scotland and will be a new role ascribed to EL facilitators in Scotland.

The aims of the study were to obtain EL facilitators’ perceptions of assessments during EL and to determine their needs to undertake CBA. This study is part of the Additional Cost of Teaching for Pharmacy (ACTp) Study which was initiated in 2018 and is described elsewhere [17]. The ACTp funding was launched by the Scottish Government in 2018 [22] to allow for protected time for EL facilitators to undertake their roles as well as be trained. These factors were not available prior to this funding [17].

## 2. Materials and Methods

In this phase of the ACTp study, we sought to obtain feedback on the adoption of CBA by facilitators during EL. Key stakeholders who will be involved in or affected by the assessment process were included in a series of studies such as students, facilitators, academic staff, and the Statutory Education Body. In this paper, we report the findings based on interviews with EL facilitators (from now on referred to as ‘facilitators’) who will be undertaking these assessments.

The methods and findings of this study are reported according to the Consolidated Criteria for Reporting Qualitative Studies (COREQ) (Supplementary S1) [23].

A series of semi-structured interviews were undertaken. Participants were recruited via a combination of purposive and snowball sampling. An invitation email along with the Participant Information Sheet (PIS) was sent by NHS Education for Scotland (NES) to all pharmacists in primary care, community pharmacy, and hospital pharmacy who had experience as an EL facilitator. Participants were also asked to suggest other potential participants. Both methods were also employed to recruit more facilitators and ensure that all three EL sites (community, hospital, and primary care) were represented. Ethical approval was granted by the Departmental Ethics Committee of the Strathclyde Institute of Pharmacy and Biomedical Sciences (EA19-13).

The interview guide was developed based on the study aims and a review of the literature. The guide sought to elicit feedback on facilitators’ perception of assessments of students during EL and to determine their needs in terms of training and resources. Face and content validation were undertaken by experts in qualitative study and pharmacy education and members of the research team. The interview guide was then pilot tested on a pharmacist who had experience as a facilitator. These responses were not included in the final analysis.

All interviews were conducted by SAJ over the phone or via Zoom, and signed consent forms were collected from all participants via email. Sessions were audio- and/or video-recorded (via Zoom), and field notes were taken to capture key points. Demographic details were also collected, and no incentives were offered to participants.

Recorded interviews were transcribed verbatim and anonymised prior to analysis. Transcripts were returned to all participants for comments and/or corrections. Results were then imported into NVivo 12 Software (QSR International Pty Ltd., Doncaster, Australia, Version 12, 2018). One transcript from each practice type (community, hospital, and primary care) was coded, and a coding map was generated. The remaining transcripts were coded, with new codes added to the coding map as and when they occurred. Thematic analysis was performed on the transcripts as well as open-ended comments, guided by Braun and Clarke’s six phase approach to coding [24]. Quotations by participants were edited on a very limited basis to remove content that did not convey meaning (repeated words, stutters) or that had no relevance to the theme being discussed. An ellipsis was used to note the removal of such extraneous content. Square brackets were used in quotations to replace sensitive or identifiable information.

## 3. Results

Fifteen facilitators participated in the interviews which were conducted from March to July 2020, five from each practice site in Scotland. While saturation of data was achieved after approximately 10 interviews, we continued until we had interviewed an equal number of participants from each of the three practice sites [25]. The majority of respondents were female (*n* = 12, 80%) and had been facilitators for less than five years (*n* = 12, 80%). When asked to rate themselves on a scale from ‘1–10′ (where ‘10′ was the highest level) on their competence and confidence in assessing students, the average response was 7.27 and 7.67, respectively (Table 1).

**Figure 1:**
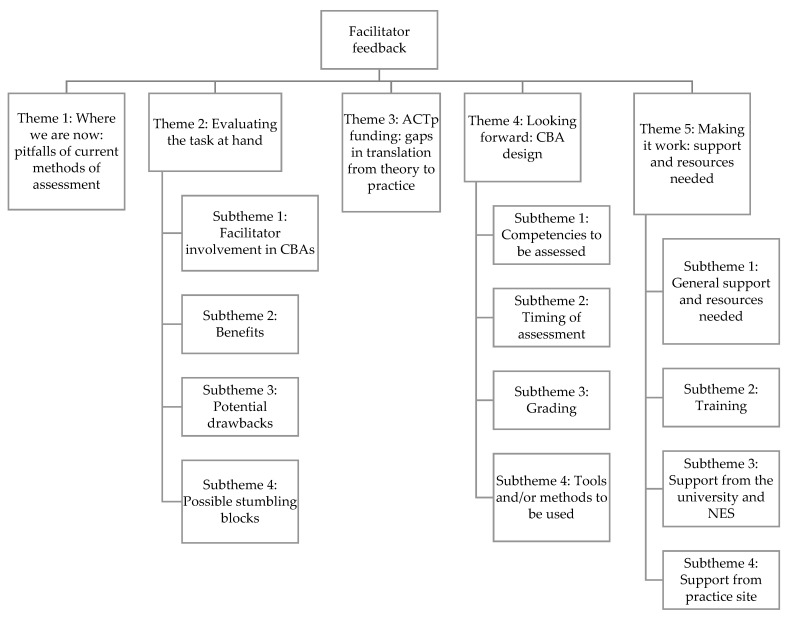
Theme and subthemes of facilitators’ feedback.

Interviews lasted an average of 37 min. The following five themes were developed from the analysis: (Figure 1 and Supplementary S2): (1) where we are now: pitfalls of current methods of assessment, (2) evaluating the task at hand, (3) ACTp funding: gaps in translation from theory to practice, (4) looking forward: CBA design, and (5) making it work: support and resources needed.

### 3.1. Theme 1: Where We Are Now: Pitfalls of Current Methods of Assessment

Facilitators felt it was *‘strange’* and *‘unfair’* that students were currently assessed by university staff who had not actually observed their performance in practice. This then did not allow for timely or accurate feedback to be provided to students on areas for improvement. Instead, assessments were currently based on what students had written in their reflective diaries, which some suggested could be manufactured. In addition, assessing the competencies that students had gained in the university via Objective Structured Clinical Examination (OSCE) sessions or role-plays were also thought to be inauthentic and not reflective of the real-world situation: *“I think it’s so different than when they’re back at uni maybe in a pretend thing. It’s really good to be able to assess them when they’re in the actual situation so it’s easy. It’s very different to speak to a fake GP or a fake doctor than it is to actually get them to go up and speak to somebody and see how they do or to speak to a patient who they know is a real patient” (H5).* Facilitators were also of the opinion that students, especially those with part-time jobs, were dismissive of their EL, as they knew they were not going to be assessed. There was some support for academics undertaking assessments, as they were more familiar with assessment procedures and criteria, and had more experience than facilitators.

### 3.2. Theme 2: Evaluating the Task at Hand

#### 3.2.1. Subtheme 1: Facilitator Involvement in CBAs

There was a general sense of anxiety and hesitation in formally assessing students, fearing that facilitators were not ready to undertake such a task due to their lack of experience with and knowledge of assessments, with one noting, *“I think pharmacists will be nervous to sign off… I think I wouldn’t feel confident being any sort of final sign off in any kind of way” (P3).* Facilitators were mainly concerned about the impact their marks would have on students’ overall grades. Facilitators were nonetheless supportive of them taking on such a role, feeling they were best-positioned to assess students as they spent a substantial amount of time observing them in the practice. This is highlighted in the following comment: *“…we are spending the majority of the time with them so we have the best knowledge to assess them because we have watched them over their week or their half days so I think it would be good for us to assess them” (C4).* It was suggested, however, that the new format should be piloted first and implemented in a phased manner and that facilitators were provided with clear guidelines and training on assessment procedures.

#### 3.2.2. Subtheme 2: Benefits

Facilitators felt that students would benefit from facilitators who were more engaged with them and *“…be assessed by someone who has actually seen them (…) doing it in a real live environment” (C3).* This would then enable students to receive real-time feedback which they can then use to identify their strengths and improve on weaknesses while still in the practice setting. It was suggested that the knowledge that they are being assessed in practice will then give students more confidence to work in the real-world setting: *“…if they know they’ve been assessed as being able to do something in real life, then they might be more confident (…) able to go out to practice and have somebody watch you and say, actually yeah, you did that really well with a real patient, maybe take some of the anxiety out for when they actually go to do their pre-reg or a placement they’re like, actually I’ve done this before I know I can do it even if it’s just once” (H5).* Facilitators also felt that students will be more relaxed, as they are not being assessed by their own lecturers and *“they don’t know us they’ll never see us again kind of thing…” (C2).*

With regard to benefits to facilitators, some felt that they would benefit from students who were more prepared, focused, and motivated to perform, as they knew they were being assessed. In assuming the new role, facilitators felt it would increase their self-development and skills in giving feedback, and encourage them to reflect on themselves, especially if students performed poorly, as illustrated here: *“…from a facilitator point of view it actually helps a facilitator gauge their own success because personally speaking, I would be devastated if I had someone with me for final year, for example, who comes with us for a week and spends a week with me…then went to assess that person and they weren’t meeting the criteria. That’s a poor reflection on you. So it allows self-assessment for the facilitator and what they’re delivering” (C3).* The new format would also ensure that feedback was more formalised and that placements were more structured, which would help facilitate the planning of it: *“…because it definitely is always easier to run a placement if it’s quite structured, it’s actually harder to run it if you’re given too much kind of freedom […] if you’ve got to focus on certain things then you can plan the placement around that” (H2).* Finally, universities would benefit from a reduction in academic workload and be able to focus their efforts in other areas to improve EL.

#### 3.2.3. Subtheme 3: Potential Drawbacks

The main drawback highlighted was the lack of standardisation and consistency in marking, given that numerous facilitators would be involved and there would be *“…a lot of variability based on level of experience, level of knowledge of how to give feedback, level of just general expertise really” (P5).* It was also thought that, by taking on such a role, this would add to the burden of responsibility on facilitators *“…without particular recognition” (P3)*. Facilitators felt that a potential drawback to students is the possibility of personality clashes between themselves and the person assessing them, which might then impact their grades.

#### 3.2.4. Subtheme 4: Possible Stumbling Blocks

Time was highlighted as a major barrier to facilitators undertaking such a role, due to their own workload, with questions on, “…*how long it would take and the input that was expected from the facilitator” (C3)*. However, this was just a matter of managing their schedule appropriately: “*Quite often when we’ve got the students, we make sure that we have the time to accommodate them so that would just be built into that. Our line manager’s’ really good at making sure that if we are taking on something that we do it to the best of our ability and the time is scheduled in for that*” *(H4)*. The short placement durations where students were only attached for half a day or a day would also impede relationship-building between facilitators and students, which was thought to be necessary to conduct the assessments. In hospitals and primary care, students are regularly attached to different facilitators other than the named facilitator. Students are also regularly supervised by other pharmacy staff such as technicians, dispensers, etc. These factors might then prove challenging when undertaking assessments, as highlighted by this comment: *“…some students might have more than one facilitator as well. So that could be difficult for them if they have to get the two facilitators together to do an assessment that might be a barrier” (H2).*

Facilitators also highlighted the fact that students will have different experiences and opportunities in different placement sites, which might impede their ability to achieve their learning outcomes: *“…there’s what 1250 community pharmacies across the country and not one of them will be the same” (C3).* However, there was an opinion that practices mostly offered a range of services which would allow students to achieve their learning outcomes, as noted by a hospital facilitator: *“So we have a surgical ward, an orthopaedic ward, general medicine ward, an acute care of the elderly, we’ve got a high dependency, there’s a paediatric, there’s a maternity, there’s an A&E…so I think that we can have huge variation of what comes through our doors and there’s always a learning opportunity” (H4).* Another barrier highlighted was with facilitators themselves, where some could be less engaged or less confident than others, which again could have an adverse outcome on the assessment process.

### 3.3. Theme 3: ACTp Funding: Gaps in Translation from Theory to Practice

Facilitators agreed that the ACTp funding allowed for facilitators to be trained on how to facilitate and supervise students, which was previously not available: *“…we’ve got this funding and the people (who) have done the facilitator training they’ve got more and more comfortable giving face to face feedback…” (C3).* It also allowed for locums or bank pharmacists to be brought in so that they had protected time with the students on placements. It was noted, however, that there was a finite supply of locum and bank pharmacists, who were also not necessarily available at the time needed or equipped with the skills needed. Despite the presence of the extra staff, facilitators still had their own workload and responsibilities, with one community facilitator saying *“…you get a couple of students so at least 4 plus your pre-reg and currently I already have to monitor staff. So it’s quite a lot of assessments to fit in so it’s not the time constraint of just getting a locum it’s also trying to find the time and yourself to split off different times for each person” (C5).* Facilitators also felt that the funding was for them to supervise and monitor students, as opposed to assessing them. There was also the perception that the funding might impact the approach to engagement with students and the assessment process, with facilitators rushing through it instead of tailoring it according to the needs of different students, as noted by a community facilitator: *“I can maybe get a student who will come in on day one and be absolutely smashing at doing everything and I could comfortably spend the rest of the day sitting with them chatting through things assessing them with no thought on time whereas I could have a student who was very unexperienced, didn’t get any of the tasks, needed constant repetition constant explanation, lengthy time dedicated to them to get them up to speed. So I would rather do that and dedicate my time to the student than trying to rush through them…to speed and then tick them off because I was getting funding to do this assessment if you will” (C3).*

### 3.4. Theme 4: Looking forward: CBA Design

#### 3.4.1. Subtheme 1: Competencies to Be Assessed

There was a near-unanimous agreement that the main competency that could be assessed by facilitators was students’ communication skills, not just with patients but also other healthcare professionals and other staff members. Other competencies identified included clinical skills such as preparing care plans, professionalism, and problem-solving skills. Some felt that the list of competencies facilitators was asked to assess should be small and that there should be some flexibility in how students achieved them, with one noting the following: *“But I think a big broad list could maybe be not effective enough because they may be jumping between tasks so maybe a bit of flexibility from the uni to say they weren’t able to complete all these tasks, but it wasn’t because they were unable as a student it was just time constraints on the student’s part” (C5).* Facilitators were also of the opinion that competencies should be individualised according to the stage students were at and the placement site, given that certain competencies were individualised to certain specialist EL sites: *“In community I was guessing some of that would be around dispensing and accuracy of that, but maybe not do that while you’re in the hospital, maybe take advantage of the other services that happened in the hospital rather than that”(P3).* Similarly, facilitators felt it should be determined which competencies might be better assessed at the university compared to practice sites.

#### 3.4.2. Subtheme 2: Timing of Assessment

There were differences in preference with regard to the timing of assessments, however most preferred for the assessment to be undertaken periodically to allow students to improve on their shortcomings, to ensure things were not overlooked, as well as to allow for extenuating circumstances such as staff absences. As students’ EL was sometimes staggered with weeks or months between two placements, this method would also be preferable to ensure facilitators did not forget how students had performed in previous placements: *“some students would come three, four five times. If they came maybe six, seven weeks apart from first to last visit you might forget what’s happened at the start…” (C5).*

Facilitators also felt that timing was dependent on the task and competency being assessed, as illustrated in the following comment: *“…the focus of the assessment for that particular placement might be communication and consultation. I think you could possibly do a formative assessment possibly towards the start of their placement and then repeat it again at the end of the placement if for example say it’s a week placement. So that I think would be useful for the student in terms of they could see a progression in themselves (…) But I think if you were assessing something like care planning then you would maybe want to do that towards the end of the placement time so they’ve got time to work on it during their placement” (P2).* It was also suggested that, due to the dynamic nature of placements and placement sites, as well as variation in student experience, there should be flexibility in the timing of the assessments: *“I think that’s probably dependent on what they’ve come to you with if they’ve had a lot of experience (…) already, they might be (…) suitable for an assessment earlier in the week” (P5).*

#### 3.4.3. Subtheme 3: Grading

There was large variation in how students should be graded, with some preferring to give scores, while others preferred rankings or percentages. There was a general sentiment of *“I think it could be quite difficult to just pass or fail” (P1).* There was indeed a general dislike for failing students, as facilitators were not comfortable doing this and felt it wasn’t *“…constructive. I don’t think it gives them the student anywhere to go with that…by failing someone you’re not giving them the opportunity to develop” (H2).* Failing a student was thought to reflect poorly on their skills as facilitators, as failure in a student would also allude to failure in their supervision. Some did agree that students should be failed if they were incompetent given the demands and importance of their future role as healthcare professionals, as long as it was aligned with the university criteria on failing: *“If they deserved it then that’s what should happen because our job is difficult it’s highly pressured, and it’s also incredibly dangerous at times. So if someone’s not competent, then they’re not competent and you can’t take a risk with patients if someone is continually incompetent” (H4).*

#### 3.4.4. Subtheme 4: Tools and/or Methods to Be Used

Most preferred using checklists to assess students for the simple reason that they were quick and easy to use. There were, however, some variations in preferences on tools to be used depending on placement sites and previous experience with a tool, i.e., those who had experience using the Mini Clinical Evaluation Exercise (miniCEX), especially hospital facilitators, preferred using it, as according to them, it allowed for more detailed feedback to be provided to students. There were also suggestions on different tools to be used to accommodate the different preferences of facilitators, the different competencies being assessed, and the different student learning styles, as highlighted by a hospital facilitator: *“I would be happy to use multiple different tools and then I think that you just find which one suits you best. Everybody learns and everybody teaches differently so different tools will suit different people… So I guess it’s about having the variation of tools out there for what works for different (people) and again it might be that that works for you as an assessor but it doesn’t really work for the student. So you have to then adapt it to the student as well as the assessor…” (H4).* There was general agreement that assessments should be undertaken face to face with students, as opposed to something completed online separately and submitted directly to the university. This was because face to face sessions would allow for students to interact with the facilitator, for feedback to be given, and for students to be able to explain their actions or inactions: *“I think face to face is better because you can have an interaction then rather than you writing down on a bit of paper and the student reading it and then the student has no ability to ask you what you mean or can you expand on that (…) it can be a much better experience for both the assessor and the student” (H4).*

### 3.5. Theme 5: Making It Work: Support and Resources Needed

#### 3.5.1. Subtheme 1: General Support and Resources Needed

There was a general call for more information to be provided on the objectives and procedures to be followed in undertaking assessments, including what was expected of facilitators in this new role. Facilitators also felt that the onus should be on universities and NES to provide training and resources to facilitators, rather than the GPhC or the RPS.

#### 3.5.2. Subtheme 2: Training

Training would be needed depending on the tools being used, such as the miniCEX, or validated tools. Similarly, facilitators required training on the procedure for assessments: *”…general guidance on how we assess the students and how we mark them. Maybe a kind of rundown of different things on what they would be doing and tasks that we would expect them to do” (C5).* Facilitators felt that peer support sessions would be very helpful, where they could see how more experienced facilitators marked students and obtain feedback on their own marking. This is captured in the following comment: *“If you then see other pharmacists and other facilitators who are marking them you’ll get kind of better overview of ‘oh that’s what they’re looking for, oh I missed that I didn’t mark them in that or oh I was being a bit too kind of strict or lenient with things’” (C5).*

#### 3.5.3. Subtheme 3: Support from the University and NES

Facilitators mainly spoke of the need for more information on students’ level of knowledge and what had been covered in the MPharm so they knew what to expect of students and were able to pitch their supervision at the right level. Similarly, clear guidance and information on the assessment criteria and procedures were needed, along with feedback on their marking, to ensure consistency and that all facilitators were marking to the same standards: *“…if it’s something you submit to the uni if you’re doing it in the right way or if you were marking if your marks were like super good compared to everyone else or super bad compared to everyone else so that you knew that you were doing okay” (H5).* Having a person to contact at the universities would also be helpful to assist facilitators when faced with challenging situations or when they had concerns about issues related to assessments. Facilitators were generally of the opinion that NES could support them in providing training and resource material, and that there should be different options for online training and resources to accommodate all facilitators: *“…I think it’s great to be able to look at those things in your own time so recorded webinars. Just again the worked examples of case-based discussion for example if people weren’t sure as to what kind of things might arise and just lots of reference material that you can look at” (P5).*

#### 3.5.4. Subtheme 4: Support from Practice Site

Facilitators generally needed support from their own practice site in matters related to the organisation of EL, such as proper staffing and time allocation to ensure they have protected time to undertake these assessments effectively. This is highlighted in the following comment from a primary care facilitator: *“…it’s not just formative assessments it’s summative assessments that are being undertaken. You can’t muck around with them so you can’t say, ‘well, I’m going to squeeze it into 15 min that I’ve taken out of my lunch break’…it needs to be adequately resourced in terms of both staff and time. (…) I think that would need to be understood (by) line managers of facilitators that that’s an integral part of the role and can’t be skimped on” (P2).* Support was also needed from other staff to supervise students, and this included nurses and other healthcare professionals.

## 4. Discussion

Facilitators in this study expressed support for undertaking this new role, noting it would benefit student pharmacists and facilitators. However, there were strong concerns about their ability to undertake assessments, with highlighted challenges and barriers such as the increased burden on their workload, their lack of knowledge in undertaking assessments, and the lack of consistency in marking. There was thus a call for adequate training to be provided about the assessment process and tools and methods to be used. While the majority agreed that competencies such as clinical, communication, and professionalism could be assessed by facilitators, a consensus could not be achieved with regard to the tools, methods, and grading of assessment, with many noting a hesitance to fail student pharmacists.

Facilitators in this study were generally anxious about undertaking this new role, mainly due to their lack of knowledge in assessments. This has been highlighted in other studies, where facilitators expressed that they hated undertaking assessments, found assessments challenging, and also questioned the accuracy of such assessments, as they only spent a brief period with students [26,27], similar to that expressed by facilitators in this study. There was also the worry about the lack of consistency in marking by facilitators in this study, again due to their lack of knowledge and experience in undertaking such a task. While some have attributed inconsistencies in marking to the lack of understanding of the assessment tools, more concrete evidence pointed to the personal attributes of the marker instead [28]. Indeed, it has been noted that facilitators are often reluctant to mark down a student, noting that they worried a bad grade could create tensions [26,27,28].

There is a culture of ‘failing to fail’ in healthcare education, where facilitators are reluctant to fail students and frequently award a pass to ‘undeserving’ students [28]. While the fear of legal percussions due to failing a student was highlighted in other studies [27,28], that was not reflected in this study. Instead, facilitators in this study felt it would reflect badly on them and their abilities, similar to that reported in systematic reviews of studies involving the experiences of dental, nursing, and medical facilitators [27,29]. While some have highlighted the importance of failing a student to ensure patient safety [27,28], facilitators in this study felt a ‘fail’ would not help a student pharmacist’s development. They also worried about the impact on student pharmacists’ progression through the course. These factors were also highlighted in other studies [27,29]. Methods proposed to address this are to train facilitators on how to address failure with trainees, instilling in facilitators a sense of obligation in the profession to fail students for the sake of patient safety, and to ensure that there are appropriate remediation pathways for students that fail. There should also be institutional support for facilitators when dealing with students who have failed [27,29]. These factors have also been highlighted by facilitators in this study, especially with regard to training and institutional support.

In adopting CBA, it is important, however, not to focus solely at identifying a lack of competence through pass/fail decisions or ranking, but to determine instead areas for improvement in competence, keeping in line with the ethos of ‘assessment for learning’ [10]. What is important is ensuring that student pharmacists receive high-quality, timely feedback based on facilitators’ observations to help them develop and achieve the required level of competence [10]. Harris et al., also noted the need to be mindful around the language used, as students who have ‘passed’ a competence think they have completed their learning, and do not realise the importance of continuing their education and training to achieve excellence—something which is key in healthcare [10]. The adoption of Entrustable Professional Activities (EPAs) might then be a more suitable method of assessment to be utilised, as they are more readily implemented and appropriate, and, instead of solely focusing on a pass/fail, they equip facilitators with justifiable grounds for ‘trusting’ a student with a task, based on different levels of ability [30,31].

Similar to facilitators in this study, feedback from facilitators in other studies have underlined a desire for an increased duration of EL, so they were able to familiarise themselves with students, and more guidance and training on how to assess students was available [26,32]. Indeed, facilitators have expressed that they lacked competence as facilitators and felt unqualified to assess students accurately [26], with one study highlighting that less than 50% of hospital facilitators agreed that they had received adequate training to be a facilitator [33]. In a study by Assemi et al., close to 50% of facilitators expressed a preference for topics on assessing students to be covered in facilitator development programmes [34]. A nationwide study of UK universities offering MPharm programmes also revealed that the topic of assessing students has not been prioritised in current training conducted by universities [35].

With the ACTp funding, all facilitators are required to undergo training under the Preparation for Facilitating Experiential Learning (PFEL) sessions conducted by NES [36]. The sessions currently include a module on teaching facilitators how to provide feedback to student pharmacists, and, as such, can also incorporate modules on the assessment process and methods, and the tools to be used. Sessions also incorporate discussion groups with other peers to allow for networking and the exchanging of ideas and experience [26,37]. This can help not only in increasing the confidence of facilitators in undertaking assessments, but also in ensuring consistency in marking. The development and training of facilitators, however, should be tailored according to the preference and needs of the facilitators, the programme, and the practice site of facilitators [26,32,34,35,38,39], all while being mindful that facilitators are adult learners, each with their own styles of learning [39]. With regard to the duration of EL, the minimum duration of an EL placement in Scotland is one week, which has increased from previous periods, which were less than a week and sometimes just for half a day [20].

Findings from this study as well as other studies have alluded to the dynamic nature of the EL, where students’ experiences are dependent on facilitators, staffing, students’ own learning styles, and prior experience, as well as placement sites—where some sites are either too busy or lack footfall [6,20,21,40]. There was a suggestion by facilitators in this study that the assessment process and learning outcomes should not be too prescriptive. Indeed, a more flexible approach has been shown to be more suitable to account for the differences in student pharmacist experience [6,41]. The quality assurance of sites through site visits and prior vetting should also be undertaken to ensure that it will allow student pharmacists to achieve the required competencies [42]. This is a function carried out by NES on behalf of the two Schools of Pharmacy in Scotland. In addition, the quality assurance of facilitators is assured, where all facilitators initially have to attend PEFL training and then an appraisal process every three years to stay on the list of NES-approved facilitators [43].

The extra burden on facilitator time and workload was noted as a cause for concern in this study, similar to that reported in other studies [26,44,45]. One suggestion to overcome this was to increase the number of placement sites [44]. In addition, the ACTp funding allows for locum or bank pharmacists to be employed to allow for protected time for facilitators in Scotland. This would help to alleviate the extra burden on facilitators. However, facilitators in this study have highlighted the lack of available locum and bank pharmacists currently due to workforce issues. Proposed methods to address this include the use of peer learning, where student pharmacists learn from more senior and experienced peers [46]. Qualitative interviews of MPharm graduates in Scotland found that participants felt they learned better when there was a Foundation Year pharmacist present [47]. This will help reduce the time spent by facilitators on teaching students [47]. CBA also traditionally relies on multiple staff, both pharmacy and non-pharmacy, to undertake the assessments of students, and, as such, the burden will not lie solely on the pharmacy facilitator [10].

### 4.1. Current and Future Research

The lack of consensus suggests that there is no one-size-fits-all approach when it comes to choosing a specific tool or method to assess students’ competencies. Instead, these should be personalised according to the respective competencies being assessed and the EL sites. Therefore, obtaining facilitators’ input on the design and structure of the assessment component will ensure that they are comfortable undertaking the tasks and using tools and methods they are familiar with. This will also ensure that resources and training programmes designed are tailored according to their actual needs, and not according to what is presumed to be of importance.

Moving forward, a community-based participatory research approach will be adopted, where researchers and the ‘community’ affected by the outcomes of future studies/plans will collaborate on all aspects of the research process through shared decision-making [48]. Thus, through a co-design process, these key stakeholders, such as students, academics, and facilitators, will be involved in the design of an assessment process which will include determining the EPAs and competencies to be assessed, tools to be used, and timelines, among other things [49]. This approach will increase ‘buy-in’ from the ‘community’ or stakeholders and give them a sense of ownership over the outcomes of the co-design process and the assessment processes that will be adopted [48,49].

Qualitative interviews have also been undertaken with facilitators and EL directors or leads from other programmes in both Scotland and England that have established practices around undertaking CBAs such as medicine, nursing, and teaching to obtain feedback and input on the design of the assessment process and this information will feed into the co-design process.

### 4.2. Limitations

All facilitators interviewed worked in Scotland. Those who volunteered for the interviews may have been more committed and passionate about undertaking their role, and this may have then influenced their responses. Although EL is increasing and evolving in Scotland in line with the GPhC Standards for IET of Pharmacists (14), currently, few pharmacists will have had much, if any, experience in undertaking the assessments of student pharmacists, and views may be from beliefs rather than from direct experience. The findings should therefore be reviewed with this in mind, given the limited knowledge on assessments and the processes involved.

## 5. Conclusions

These findings provided valuable insights which will enable the development of suitable assessment tools for use during EL. Such assessments will be increasingly important across Great Britain as part of the MPharm degree regulated by the new GPhC Standards. The opinions of students, assessors, and other stakeholders in this and other studies will be considered when selecting existing assessment tools and devising new ones. Induction and training will also be developed and evaluated, so that all those involved are confident about the concepts and processes. The intended outcome is the development of valid and relevant learning and assessment for students preparing for increasingly complex clinical roles in practice.

## Figures and Tables

**Table 1:** Demographics of interview participants (*n* = 15).

Demographics	*n* (%)
Age	
20–29	7 (46.7)
30–39	4 (26.7)
40–49	3 (20.0)
50–59	1 (6.67)
Education *	
BSc	3 (20.0)
MPharm	13 (86.7)
Postgraduate masters	4 (26.7)
Ph.D.	1 (6.67)
Number of years registered as a pharmacist	
<5	4 (26.7)
5–9	6 (40.0)
10–14	1 (6.67)
15–19	1 (6.67)
20–24	2 (13.3)
25–29	1 (6.67)

* Respondents were allowed to select more than one option; therefore, totals might exceed 100%.

## Data Availability

The data presented in this study are available on request from the corresponding author. The data are not publicly available due to privacy.

## Supplementary Materials

The following supporting information can be downloaded at: https://www.mdpi.com/article/10.3390/pharmacy10040090/s1, Supplementary S1: COREQ (COnsolidated criteria for REporting Qualitative research) 32 item Checklist [23]. Supplementary S2: Themes, subthemes, and illustrative quotes from facilitator interviews.
