# Supplementary material for: Competency-Based Assessment in Experiential Learning in Undergraduate Pharmacy Programmes: Qualitative Exploration of Facilitators’ Views and Needs (ACTp Study)
Source: Pharmacy (Basel). 2022 Jul 26;10(4):90. doi: 10.3390/pharmacy10040090 (PMC9332294; doi:10.3390/pharmacy10040090)
Supplement: Supplementary file 1 [file pharmacy-10-00090-s001.zip › pharmacy-1774446-supplementary.pdf]

Supplementary S1

COREQ (COnsolidated criteria for REporting Qualitative research) 32 item Checklist Tong A, Sainsbury P, Craig J. Consolidated criteria for reporting qualitative research (COREQ): a 32-item checklist for interviews and focus groups. International Journal for Quality in Health Care. 2007. Volume 19, Number 6: pp. 349–357.

Domain	Comment	Section Reported in or Not Applicable (N/A)
Domain 1: Research team and reflexivity		
Personal characteristics		
1. Interviewer/facilitator	SAJ, a research associate, conducted the interviews.	Methods
2. Credentials	The research team comprised SIX academic pharmacists (SAJ, TJ, JP, AP, SC, ACB) with doctoral degrees.	N/A
3. Occupation	SC is a Professor of Pharmacy Education and Practice and Academic Strategic Lead for Clinical Practice, ACB is a Professor of Learning and Teaching and Director of the MPharm programme, AP is Associate Postgraduate Pharmacy Dean at NES, JP is an external consultant employed by NES to oversee the study, and both SAJ and TJ are research associates.	N/A
4. Gender	One male-identifying and five female-identifying researchers. The interviewer was female-identifying.	N/A
5. Experience and training	All six research team members had experience in the design and analysis of qualitative research, as well as studies focusing on pharmacy education.	N/A
Relationship with participants		
6. Relationship established	AP may have interacted with some of the participants in her role in NES but was unaware of the identity of any participant. ACB may also have interacted with the participants in her role as programme director but was also unaware of the identity of any participant. All other researchers had no previous interactions with the participants.	N/A
7. Participant knowledge of the interviewer	Interviewer credentials were available on the participant information sheet handed out to participants. Prior to the start of the interviews, the interviewer informed participants about the objectives of the study and answered any questions participants may have had about the study as well as those involved in it.	N/A
8. Interviewer characteristics	ACB, JP, AP, and SC had no EL in their undergraduate period, while SAJ and TJ underwent EL during their undergraduate period outside the UK. SAJ did have previous experience outside the UK supervising students during their EL.	N/A
Domain 2: Study design		
Theoretical framework		
9. Methodological orientation and theory	A descriptive qualitative research design was used.	Methods
Participant selection		
10. Sampling	Purposive and snowball sampling were used.	Methods

11. Method of approach	Participants were invited via email sent out by NES and all via snowball sampling by participants.	Methods
12. Sample size	15	Results
13. Non-participation	None.	N/A
Setting		
14. Setting of data collection	Interviews were conducted via Zoom or over the phone.	Methods
15. Presence of non-participants	None.	N/A
16. Description of sample	Fully presented in results section.	Results
Data collection		
17. Interview guide	An interview guide was developed based on the study objectives and validated by experts in qualitative design and health education.	Methods
18. Repeat interviews	None.	N/A
19. Audio/visual recording	All sessions were audio recorded.	Methods
20. Field notes	Field notes were captured during the sessions to capture key points and make notes on any perceptions with regard to participants' responses.	Methods
21. Duration	Individual interviews lasted an average of 37 minutes.	Results
22. Data saturation	Data saturation was achieved after 10 interviews; however, sampling continued until there was equal representation from each practice site.	Results
23. Transcripts returned	Yes.	
Domain 3: analysis and findings		
Data analysis		
24. Number of data coders	SAJ coded the data. A coding map was generated which was discussed with the principal investigator, ACB, and then used to code the remaining transcripts. Any discrepancies were resolved by consensus.	Methods
25. Description of the coding tree	A description of the coding tree is not provided. Only themes and subthemes generated are presented.	
26. Derivation of themes	Themes were derived from the data.	Methods
27. Software	None.	
28. Participant checking	Not done.	
Reporting		
29. Quotations presented	Yes, quotations are presented. Anonymized participant details are used for each quotation.	Results
30. Data and findings consistent	Yes, there is consistency between data and findings.	Results
31. Clarity of major themes	Major themes are clearly illustrated.	Results
32. Clarity of minor themes	Minor themes are also highlighted.	Results

Supplementary S2

Themes, subthemes, and illustrative quotes from facilitator interviews.

**Themes**	**Elaboration #**	**Illustrative Quotes ***
**Theme 1: Where we are now: pitfalls of current methods of assessment**		
	Unable to guide students to improve in a timely manner.	*"So it's quite hard when you're not the ones assessing them to make sure that they're doing the right things as well, or getting to see the right things" (H5)*

	Current assessments perceived as inauthentic and based on contrived situations.	*"I mean anybody can go back and write a good essay…that they've researched but they might not be performing in the way that they write that down afterwards. You can write what you think you should have done, but you might not have actually done it." (H2)*
	Perception that students take it less seriously as they know they are not going to be assessed.	*"…and they can be quite dismissive of it they obviously think that because they work on a Saturday (…) they do all this stuff it's all fine and dandy, they don't need to give it a lot of their attention and have real dedication to it so I suppose that's probably the big downside of not actually assessing them in practice." (C3)*
	Academics are more familiar with assessments.	*"…when they're being assessed back at the university, I suppose, at least it's somebody who is experienced with assessment and somebody who is I suppose they're assessing similar competencies because obviously lots of people will have had a different experience in practice." (P5)*
**Theme 2: Evaluating the task at hand**		
Subtheme 1: Facilitator involvement in CBAs	Overall support expressed in undertaking the role	*"…it's better if the assessment comes directly from us… I think moving forward it is a much better idea for us to be able to give feedback to the students rather than us feeding back to the university and then the university having to feed it just seems like an extra step that is not required." (H4)*
	Should be a phased roll-out with a trial run prior to establishing it formally.	*"…at the start, it might be good that we don't assess them…we will carry out an assessment but that wouldn't have any effect on the student so it will be almost like a practice run for ourselves and even a practice run for the students…"(C5)*
Subtheme 2: Benefits	Students will benefit from facilitators who are more engaged.	*"… more dedicated and more interested in the students and their development."(P4)*
	Students will get immediate feedback which will allow them to identify their strengths and weakness and improve on them in the practice setting.	*"…they could get sort of more real time feedback…some of them they don't actually assess the skills until the OSCEs at the end of the year well it's a bit late isn't it if there's an actual problem for example with their communication skills that they maybe don't even realize that they have, then you want to give them the chance to fix it and you want to give them a chance in the environment (…) to practice it if they need to… there's no point in letting them fail and then telling them six months later that they can't do it when they've missed the opportunity to practice it."(H2)*
	Facilitators will benefit from students who are more engaged and motivated to perform.	*"…if they were knowing at the start of it that there is going to be some sort of assessment I think they would turn up and come a bit more prepared to a kind of experiential learning. I know a lot of them feel at the moment that they are just used as an extra labourer. A lot of them already have Pharmacy jobs so they're coming in and they're being asked to do certain tasks and they've already done them in quite a lot of their work and then they've got to go back and do a diary entry on something that they've already done before. So I think if they were to come in and know that at the end of it they don't just need to write up something they actually need to prove to the person in front of them that they know what they're doing I think that would be very valuable." (C5)*
	Being tasked with assessing students will motivate facilitators to be more committed to the	*"…we'll make the placement work better if we're given the opportunity in a structured way to feedback to people and tell them how they can improve, if you can explain why you're doing things a certain way because you need to meet these competencies and you're not quite there*

	placement and focused on making it a good experience for students, which they felt was a benefit to themselves.	*yet, but we're going to help you so yes I think it would probably be quite beneficial for us as well." (H2)*
	Facilitators felt this would ensure feedback was more formalised and that placements were more structured.	*"…we had a difficult student and I think if we had maybe been able to feed back to her that maybe her attitude wasn't great or maybe asked her what she would find more interesting, I think maybe we would have had a better experience with her rather than it kind of carrying on and carrying on and us not really being able to do anything about it." (H4)*
Subtheme 3: Potential drawbacks	Lack of consistency in marking due to lack of experience with assessment.	*"we're probably more variable than the uni tutors probably because that's not what we do all day. We maybe have them a few times a year or once a year so we're maybe less consistent with our marking." (H5)*
	A drawback to students is the potential personality clash with the person assessing them.	*"…if they take a dislike to us or they don't think it's totally fair that somebody they haven't met before is giving feedback on them..." (C2)*
Subtheme 4: Possible stumbling blocks	The limited placement duration was another key barrier as facilitators would not be able to build a relationship with students and properly assess them.	*"Where it would prove more difficult would be when you're only seeing the younger students for half a day and then they disappear for a few weeks. That wouldn't work the same I think they would have to be in for a full week as well. You couldn't just come in for a half day, I can't see that working. I can't see us being able to assess them. They would need to have an extension." (C4)*
	Students are attached to different facilitators and different staff during placements which could impact the assessment process.	*"So as a facilitator (…) I'm not necessarily with the student 24-7…so it's recognizing that there is going to be more than one pharmacist in our setting that will be associated and involved with the learning of that and the experience of that student, just to give them as rich an experience as possible really." (P5)*
	Students have different experiences in different placement sites, with some not being able to achieve all their learning outcomes in certain sites.	*"…the situation that they come into in the pharmacy it may be a really, really busy day, your staff might be kind of all over the place, you might be all over the place so I think maybe based on a kind of certain time it might not be great it might not be the best."(C5)*
	Facilitators who were less confident or less committed could have an impact on the assessment process.	*"…people are going to be more up for it and probably some pharmacists maybe aren't as hands-on and that could impact on the students' marks." (C4)*
**Theme 3: ACTp funding: gaps in translation from theory to practice**		
	There was, however, a finite supply of locum or bank pharmacists, and replacements may not have the level of expertise needed.	*"So I've still only got a finite supply of pharmacists even with the ACT funding. It's very difficult to find extra pharmacists' time (…) I can't get a locum in for a week or two while I've got a student in primary care that's just not practical….so I need somebody else who can also do that and there's a very limited expertise there, a limited body of that people will have all types of expertise." (P5)*
	Funding thought to serve different purpose, other than assessing students.	*"Well the ACT money has obviously become available at the moment when we're not assessing the students. So it's became available to give us time to dedicate to (the) student not to assess the student." (C3)*

**Theme 4: Looking forward: CBA design**		
Subtheme 1: Competencies to be assessed	Competencies mainly focusing on communication skills, as well as professionalism, problem-solving, and clinical skills, could be assessed by facilitators.	*"so I would definitely think communication, professionalism… we've got certain facilities in university but again that's a very kind of fake set up environment whereas we've got it all at hand in the pharmacy so you would be able to see how they would be able to act, see how they would interact with also other staff members, not just patients, but how they would be in a group setting." (C5)*
	Competencies to be assessed dependent on placement site and stage students are at.	*"I think when it comes to maybe the lower years when they're starting to do their kind of counter selling and things like that, the WHAMM questions…in the higher up years when they are maybe a bit more involved, one on one with people." (C2)*
	Certain competencies might be best assessed at the university versus the placement site.	*"…certain competencies are better assessed in a university setting, but I think a lot of the competencies could be assessed in a pharmacy and we would be in a great position to assess a lot of them..." (C5)*
Subtheme 2: Timing of assessment	Assessments to be undertaken periodically instead of leaving at the end to ensure students can improve on their weaknesses during the placement.	*"I think it would have to be done periodically, because I think it's unfair to get to the end and tell someday that they've not achieved something (…) if they are failing in something that we give them a chance to remedy that before the end of it." (C1)*
	Assessments undertaken only at the end to allow students to improve on weaknesses.	*"I think at the end would probably be better because if you're going to give them feedback as you go then at the end would be better to give them time to improve along the way if you've got say a week or so. So also to give them a chance to improve if they're performing quite poorly before actually putting pen to paper kind of thing." (C2)*
	There should be flexibility in timing, as opportunities to achieve learning outcomes could vary depending on placement site and students' level of experience.	*"…you don't want to put too much pressure on the situation because sometimes just how it happens with the patients that are available, who you can see, what sort of experience that you can give them will depend on the staffing, other people who are sick in the department, or who turns up to some of the clinics and things." (P3)*
Subtheme 3: Grading	Variation in how students should be graded, with some preferring to give scores, ranking, or percentages, and few agreeing with giving a pass or fail.	*"Probably I was thinking it would be towards like a ranking so if I was thinking of the case discussions or the miniCEX forms that we use for Foundation maybe it's a meets expectations, exceeds expectations or whatever the rest of them are and with the proviso that you're expecting less of them because they're a student..." (P3)*<br>*"It's probably easier to pass or fail them, I'd probably feel more confident passing or failing them unless I had like really, really clear guidance on how to score them because I think I would feel worried that I was maybe scoring them badly, and it would affect their grade or something unfairly…"(H5)*
	Failing a student would reflect poorly on facilitators as it suggested a failure in their supervision as well.	*"…your responsibility to try and explain to them how not to do it. So if you did fail a student then I think that would be a reflection on you as well and I think at some point, you should have stepped in to try and help them." (C5)*
	Some agreed students should be failed if they	*"…if I could fail somebody but if it were something where I could show them like you've not met this criteria, then I think I'd be okay." (H5)*

	were incompetent, as long as it could be backed up by university criteria on failing.	
Subtheme 4: Tools and/or methods to be used	Preference for checklists to be used as they were simple and quick.	*"I suppose the end goal's got to be, do you sign them off as a yes or no so it's back to the black and white thing. So I suppose a checklist is probably the easiest way to log that, diarise it and then come to a decision with it." (C3)*
	MiniCEX preferred by those with prior experience, as it allowed more detailed feedback to be provided compared to checklists.	*"…miniCEX I suppose that would give them a focus just that would be certainly a bit more time consuming but it might be more useful for the students they'd maybe get more feedback from that." (H2)*
**Theme 5: Making it work: support and resources needed**		
Subtheme 1: General support and resources needed	Information needed on objectives and the assessment process, including expectations of facilitators.	*"So if a student comes in and they aren't maybe doing a task at what point have we to step in or have to leave it to the student and let them do the task and we assess them without any input or (…) are we allowed to input and say, 'look, this isn't really how you do it, this is how you would improve that'? So kind of guidance on what's expected of us and what we're allowed to do." (C5)*
Subtheme 2: Training	Training needed on assessment procedure and tools to be used to undertake assessments.	*"…depends what form of assessment you're going to use but you need training on the specific tool that you wanted us to use, like if it was miniCEX then you would have to train everyone to ensure they were following the same standards." (H2)*
Subtheme 3: Support from the university and NES	Information needed on students' level of knowledge as well as expectations.	*"…so it would be really quite useful to get a bit of a course outline of where they are, what competencies they've already marked off with the university, what the university feel they have already covered, and what they're going to go straight on to when they come back because then it makes it relevant for the students. It's always easier to practice something that you've just been taught, makes it much more relevant." (H2)*
	Information on performance in previous attachments would assist in carrying out assessments.	*"…there might be great comments and you're like 'oh ok I know what to expect of the student', then they come in and they're not quite what they live up to. So I think that could be dangerous, potentially, but I also think knowing something about the student that you've got coming in might be of benefit to assess them." (C5)*
	Clear guidance and information on assessment criteria and procedures also needed to ensure consistency.	*"Again I think if you are marking them according to their marking grids, then you should be given their marking grids and have those marking grids explained to you so that there's little to no variation in the marking between the different assessors." (H4)*
	Contact person at the universities needed to assist with issues that facilitators might find challenging.	*"Probably just a contact either at the uni…so that if there was something you weren't sure of you could phone and you weren't having to wait till after the placement or something (…) if you didn't know how to mark them or there was an incident and you didn't know how to deal with it (…) that you can easily contact someone and just check with them." (H5)*
Subtheme 4: Support from practice site	Support mainly in ensuring protected time by hiring locum or banks pharmacists.	*"…so getting the sort of green light from the company to get locums in and utilise that funding I think it would be important. You don't want to be worrying about the shop and worrying about the patients whilst also you're deciding the mark of a student, deciding their fates how*

		they get on. So from the company itself and the pharmacy it would be utilizing that money to get the support in to allow us to carry out these assessments." (C4)
	Support also needed from other staff to supervise students.	*"…but I think it's important that you do have everybody on board or else the student's not going to have a good experience because…they can't shadow you for the whole week they need to be with other people so you need to be sure that other people are going to be willing to take them."(H2)*

miniCEX: Mini Clinical Evaluation Exercise; NES: NHS Education for Scotland; GPhC: General Pharmaceutical Council; RPS: Royal Pharmaceutical Society. * H: hospital, C: community, P: primary care.
